# Dual-expression-site fowl adenovirus 4 (FAdV-4) with high viral titer: a promising viral vector for multivalent poultry vaccines

**DOI:** 10.1186/s13567-026-01820-z

**Published:** 2026-08-13

**Authors:** Zhihui Tang, Wenjian Liu, Biao Xu, Dengfei Feng, Shuhui Liu, Wentao Fan, Suquan Song, Liping Yan

**Affiliations:** MOE Joint International Research Laboratory of Animal Health and Food Safety, Jiangsu Engineering Laboratory of Animal Immunology, Jiangsu Detection Center of Terrestrial Wildlife Disease, Institute of Immunology, College of Veterinary Medicine, Nanjing Agricultural University, Nanjing, 210095 Jiangsu China

**Keywords:** Fowl adenovirus serotype 4, multivalent vaccine vector, foreign gene expression

## Abstract

Fowl adenovirus serotype 4 (FAdV-4) is a nonenveloped double-stranded DNA virus with a 43–45 kb genome, making it a promising viral vector for developing multivalent poultry vaccines. However, limited genomic studies have hindered the design of strategies for generating recombinant FAdV-4 capable of expressing multiple foreign proteins while maintaining high viral titers. In this study, three deletion mutants (rD14AB, rD2042, rD19A) were generated to assess their roles in viral replication and pathogenicity. Only rD2042 showed impaired replication and virulence compared with wild-type FAdV-4. Foreign gene expression assays demonstrated that the D14AB site had a maximum insertion capacity of 2349 bp, and an SV40 poly(A) signal was required for efficient protein expression and viral propagation at this locus. In contrast, the D19A site tolerated insertions of up to 1257 bp in the absence of this signal. Using the insertion strategy optimized for wild-type CH_JS_2017, we modified the previously reported nonpathogenic rON1 strain (harboring the Hexon gene from the attenuated ON1 strain) to co-express mCherry, eGFP, and mTagBFP2 at the two independent sites. SPF chicken trials verified the safety of the engineered virus at immunization age and the genetic stability of exogenous genes. The recombinant virus maintained wild-type-comparable titers while carrying 3606 bp of total foreign sequences, highlighting its dual-site advantage and potential as a multivalent poultry vaccine vector.

**Supplementary Information:**

The online version contains supplementary material available at 10.1186/s13567-026-01820-z.

## Introduction

With the development and application of gene-editing technologies, various recombinant viral vectors expressing foreign proteins have been widely explored for gene delivery, targeted therapy, and vaccine development, including adenoviruses [1–3], poxviruses [4, 5], herpesviruses [6–8], and Newcastle disease viruses [9–11]. Human adenoviruses are the most widely used viral vectors for gene therapy [12] and the development of recombinant vaccines [13]. Based on mature human adenovirus vector experience, researchers are exploring the modification of other species’ adenoviruses into vaccine vectors, among which Fowl adenovirus serotype 4 (FAdV-4) is a highly promising and widely noted candidate [14–18].

FAdV-4 can infect various avian species [19], giving it broader prospects than human adenoviruses for multivalent poultry vaccines. Moreover, FAdV-4 causes hepatitis-hydropericardium syndrome in broilers, with a mortality rate ranging from 30 to 100% [20]. Its high pathogenicity further highlights the value of developing FAdV-4 into a vector—such a vector could simultaneously deliver foreign antigens and induce immunity against FAdV-4 itself. This pathogen is globally prevalent and poses a significant threat to the poultry industry; this virus has caused widespread epidemics and severe economic losses in China’s poultry industry since 2015 [21]. Therefore, developing FAdV-4 into a viral vector can both deliver exogenous genes and protect against FAdV-4, thus simplifying immunization procedures and breeding costs.

Similar to human adenoviruses, FAdV-4 belongs to the *Adenoviridae* family and has a nonenveloped, double-stranded DNA genome of 43–45 kb [22], which is larger than that of human adenoviruses, thus potentially allowing for the insertion of more exogenous gene fragments. In the well-established human adenovirus vector system, the E1 region is essential for viral replication whereas E3 region is nonessential; with E1-expressing packaging cell lines, E1/E3-deleted vectors typically accommodate approximately 4.5–8 kb of foreign DNA while improving vector safety [23, 24]. This concept provides useful guidance for engineering FAdV-4-based vectors. To date, most studies have examined the effects of individual ORF deletions on viral replication and pathogenicity to identify potential insertion sites for foreign-gene expression [14, 25–27]. However, single ORF deletion has notable limitations: it only provides limited insertion capacity, and most resulting recombinant vectors still exhibit impaired replication and reduced titers relative to wild-type viruses [28–30], a common defect also observed in recombinant FAdV-4 [18, 26, 31]. This problem restricts the field application of recombinant viruses and increases production costs. Thus, developing FAdV-4 into a viable vaccine vector requires identification of optimal insertion sites that allow robust foreign gene expression while maintaining normal viral replication and titers. Given this challenge, guided by available FAdV-4 transcriptomic annotations [32], combined deletion of adjacent ORFs under a shared promoter within nonessential genomic regions far from replication-essential genes can effectively expand exogenous insertion capacity, preserve viral replication ability, and facilitate broader applications of FAdV-4 in multivalent vaccine development.

To construct attenuated FAdV-4 vaccine vectors possessing high foreign gene insertion capacity while maintaining robust viral replication titers, we first constructed novel insertion sites via combined deletion of adjacent nonessential ORFs based on the wild-type FAdV-4 CH_JS_2017 strain. A series of assays identified two ideal sites, D14AB (ORF14/14A/14B deletion) and D19A (ORF19A deletion), which maintain normal viral replication and support stable foreign protein expression. Based on our previously published work [33], we employed the attenuated rON1 strain with previously confirmed safety in SPF chicken, constructed via hexon replacement between the wild-type CH_JS_2017 strain and the ON1 strain, as the vector backbone. Fluorescent protein cassettes were inserted into the validated sites to produce recombinant viruses, confirming the practical applicability of these sites in attenuated live vaccine vectors. In addition, by exploiting the replication-enhancing effect of ORF19A deletion (or substitution with reversely inserted expression cassettes), the recombinant virus can coexpress three fluorescent proteins, and maintains viral titers comparable to those of the wild-type CH_JS_2017 strain. Collectively, these findings provide a solid foundation for the development of FAdV-4-based multivalent avian vaccine vectors by achieving both high foreign gene insertion capacity and robust replication titers.

## Materials and methods

### Cells and virus

The Leghorn Male Hepatoma (LMH) cell line was purchased from ATCC (CRL-2117) and used within 20 passages for virus rescue and passage. LMH cells were cultured in Dulbecco’s Modified Eagle Medium/Nutrient Mixture F-12 (DMEM/F12; Gibco, USA) containing 10% fetal bovine serum (FBS; Gibco, AU) at 37 °C with 5% CO_2_. Wild-type highly virulent FAdV-4 strain CH_JS_2017, isolated and preserved in our laboratory, served as the parental strain for infectious clone construction in this and prior studies [33]. Its complete viral genome sequence has been submitted to GenBank with the accession number OR584077.1. The rFAdV-4 strain and artificially attenuated rON1 strain with Hexon gene editing were rescued using an infectious clone [33].

### Plasmids and bacteria

The plasmids pRed (expressing Redα, Redβ, and Redγ) and pUC19-rpsL-KanR (containing counter-selection markers) were included in the Counter Selection BAC modification kit (Gene Bridges, Germany). FAdV-4 infectious clone pBAC-FAdV4 (pFAdV4) was constructed previously [33]. All plasmids were stored and propagated in *Escherichia coli* DH10B (Biomed, China), then purified using the TIANprep Mini Plasmid Kit (Tiangen Biotech, China). Fluorescent protein expression cassettes were generated by overlap PCR and ligated into linearized plasmid pMD18-T for storage (TaKaRa, Japan).

### Polymerase chain reaction (PCR)

Primers were listed in Additional file 1, and synthesized by Sangon Biotech, China. All fragments used for the construction of recombinant infectious clone were amplified by high-fidelity PCR using 1 × Hief Canace® Plus PCR Master Mix (Yeasen Biotech, China). Positive clones were identified by regular PCR using 1 × Hief® PCR Master Mix (Yeasen Biotech, China). PCR conditions were as follows: initial denaturation at 95 °C for 5 min; 30 cycles of denaturation at 95 °C for 10 s, annealing at 60 °C for 15 s, extension at 72 °C for 30 s per kb; and a final extension at 72 °C for 5 min. Schematic diagrams of these cassettes are shown in Additional file 2. Sequences of expression cassettes were provided in Supplementary Information.

### Recombinant FAdV-4 construction

Gene-edited infectious clones were generated by two-round recombination mediated by pRed-origin proteins in DH10B, based on pFAdV4. Procedures were in accordance with the manufacturer’s instructions of the Counter Selection BAC modification kit with electroporation conditions adjusted to 1500 V, 25 μF, and 200 ohms in a 1 mm electroporation cuvette (Bio-Rad, USA). For the second round recombination, to enhance recombination efficiency, the length of homology regions (HR) was extended to hundreds of base pairs. Positive clones were further verified by PCR and Sanger sequencing (Sangon Biotech, China).

Recombinant infectious clones were digested with PacI (Yeasen Biotech, China) and then transfected into LMH cells for virus rescue [33]. Linearized plasmids were transfected into cells using Hieff Trans® Liposomal Transfection Reagent (Yeasen Biotech, China) following the manufacturer’s instructions.

### Recombinant FAdV-4 virus titration

Viral stocks were harvested when cytopathic effects (CPE) reached 70%–80% or after the designated incubation period. Following three freeze–thaw cycles, samples were centrifuged at 12,000 × *g* for 10 min to eliminate cellular debris, and viral supernatants were collected.

LMH cells were seeded into 96-well plates, and viral Tissue Culture Infectious Dose 50 (TCID_50_) titration was performed once cells reached approximately 80% confluence. Viruses were serially diluted tenfold in DMEM/F12 medium containing 2% FBS, with dilution gradients ranging from 10^−3^ to 10^−10^. Eight replicate wells were set for each dilution, and 100 μL diluted viral suspension was added into each well. After incubation at 37 °C with 5% CO_2_ for 72 h, viral TCID_50_ titers were calculated using the Reed–Muench method [34]. All assays were conducted in triplicate.

### Effects of combined nonessential ORF deletion on in vitro replication of recombinant viruses

To investigate the effects of simultaneous deletion of nonessential ORFs on viral in vitro replication fitness, one-step growth kinetics of recombinant viruses were determined. To maintain stable viral biological phenotypes, rescued gene-deleted strains were serially passaged for 5 passages in LMH cells. Then, the recombinant viruses were inoculated into LMH cell cultures at a multiplicity of infection (MOI) of 0.05 in 6-well plates. Supernatants were collected at 24, 36, 48, 60, 72, and 96 h postinfection (hpi). Viral titers at each time point were detected to construct viral one-step growth curves. Meanwhile, cellular CPE was observed and photographed under a fluorescence microscope at 24 h intervals.

### Real-time qPCR quantification of viral genome copy numbers

To further evaluate genomic DNA replication and proliferation capacity of recombinant viruses, FAM-labeled TaqMan probe real-time quantitative PCR was performed to absolutely quantify viral genomic DNA copies in cell supernatants harvested at serial time points postinfection, and viral one-step growth curves were subsequently plotted.

Viral DNA was extracted with viral DNA/RNA extraction kits (Vazyme Biotech, China). An identical volume of viral supernatant was used for nucleic acid extraction across all samples, and the purified viral DNA was eluted in a final volume of 53 μL. qPCR amplification was performed in a total 20 μL reaction system using AceQ qPCR Probe Master Mix (Vazyme Biotech, China) in strict accordance with manufacturer instructions, and 1.2 μL template DNA was uniformly loaded into each reaction. The qPCR thermal cycling conditions were as follows: initial denaturation at 95 °C for 5 min, followed by 40 cycles of 95 °C for 10 s (denaturation), and 60 °C for 30 s (annealing and extension). Primers and TaqMan probes targeting the viral Hexon gene are listed in Additional file 1, with an amplified fragment length of 216 bp.

Absolute quantification standard plasmids were prepared by recovering the target amplified fragments and ligating them into the pCE3 vector (Vazyme Biotech, China) via TOPO cloning. The total length of the constructed recombinant standard plasmid was 2014 bp. Serial diluted standard plasmids (ranging from 10^2^ to 10^9^ copies/μL) were used to establish the linear standard curve, and the regression equation was as follows:

$${\text{Ct value = }}\,{ - 3}{\mathrm{.1573}}\, \times \,\log_{10} \,\left( {{\mathrm{copy}}\,{\mathrm{number}}} \right)\, + \,39.949\,\left( {R^{2} = 0.9938} \right)$$

The absolute copy number of standard plasmid was calculated using the universal formula:

$${\mathrm{Plasmid}} {\mathrm{copy}} {\text{number }}({\mathrm{copies}}/\mu {\mathrm{L}}) = \left[ {{6}.0{2} \times {1}0^{{{23}}} \times {\text{plasmid concentration }}\left( {{\mathrm{ng}}/\mu {\mathrm{L}}} \right)} \right] \, / \, ({\text{Total plasmid length}}\left( {{\mathrm{bp}}} \right) \times {66}0)$$

PCR amplification efficiency calculated from the curve slope was 97.4%, which conforms to the reliable quantitative range of TaqMan real-time qPCR. The quantitative range of the standard curve established in this study corresponded to Ct values from 11.56 to 33.39. Any amplification signal with a Ct value > 35 was classified as assay background, and the corresponding sample was deemed negative for viral DNA. This threshold was conservatively selected, as the quantitative range of the standard curve ended at Ct 33.39, and sporadic amplification signals in no-template controls (NTCs) were exclusively observed at Ct values above 35. To eliminate exogenous nucleic acid contamination, NTCs were included in each batch of experiments.

### Effects of combined nonessential ORF deletion on in vivo replication of recombinant viruses

To investigate the effects of simultaneous deletion of nonessential genomic ORFs on viral in vivo replication fitness, 21-day-old specific-pathogen-free (SPF) chickens were challenged to evaluate the pathogenicity of recombinant viruses. A total of 75 chickens were randomly divided into five groups (15 chickens in each group); one control group and four treatment groups. Chickens were housed in isolators and had free access to feed and water. The temperature, humidity, and light conditions were identical in all isolators.

Chickens from treatment groups were inoculated with 0.3 mL (10^6.5^ TCID_50_/0.1 mL) rFAdV-4, rD14AB, rD19A, and rD2042, respectively, via intramuscular injection. Chickens from the control group were intramuscularly injected with 0.3 mL DMEM/F12. To ensure reliable mortality and morbidity data, 15 challenged chickens per group were randomly divided into two functionally distinct subgroups. The first subgroup (*n* = 10) was continuously observed for mortality and clinical manifestations for 7 days without any intervention. All morbidity and mortality data in this study were derived exclusively from this subgroup. The second subgroup (*n* = 5) was dedicated to early virological and pathological detection. At 2 days postchallenge (dpi), dead birds were collected, and surviving individuals were euthanized via CO_2_ asphyxiation for tissue sampling. Specimens from this subgroup were applied for organ viral load quantification and histopathological analysis, and this group was excluded from mortality statistics.

Tissues including the heart, liver, spleen, kidneys, and ileum were harvested from the second subgroup. A portion of heart, liver, and kidney tissues was fixed in 4% neutral formalin buffer at room temperature for 72 h. All collected tissue samples were stored at −80 °C. Fixed tissues were then embedded in paraffin, sealed, and cut into 5-µm sections. The sections were mounted on glass slides and stained with hematoxylin and eosin (H&E) for further histopathological examination.

Meanwhile, to further evaluate in vivo replication of the viral strains in SPF chickens, viral genome loads in heart, liver, spleen, kidney, and ileum were quantified using the previously validated qPCR method. To further eliminate viral genome copy number variations caused by tissue sample weight differences during sampling, tissue samples were weighed, homogenized in 1 mL PBS, and centrifuged at 12,000 × *g* for 10 min. A 333 μL aliquot of the homogenate supernatant was used for viral DNA extraction, with a uniform elution volume of 53 μL for all samples. Approximately 1.2 μL of the eluate was used for viral genome copy number quantification. Copy numbers were calculated using the aforementioned standard curve equation, converted to the corresponding viral genome copies in 1 mL of tissue homogenate, and normalized to the weight of each individual tissue sample to obtain the final unit of copies per milligram (copies/mg).

### Exogenous gene expression strategies at distinct insertion sites

To investigate exogenous protein expression strategies at three genomic sites: D14AB (combined deletion of ORF14, ORF14A and ORF14B), D19A (ORF19A deletion), and D2042 (combined deletion of ORF20 and ORF42), previously constructed fluorescent protein expression cassettes were inserted into the above sites to rescue recombinant viruses.

To identify suitable insertion sites and exogenous gene expression strategies, we further assessed fluorescence expression and growth properties of recombinant viruses stably maintained for five serial passages. These recombinant viruses were inoculated into six-well plates seeded with LMH cells at a confluence of 80%–90% at an MOI of 0.05. Fluorescence images were collected at 24, 36, 48, 60, 72, and 96 hpi. Throughout image acquisition, unified parameters of the inverted fluorescence microscope were strictly controlled: 10 × eyepiece, 10 × objective lens, 1-s exposure time, and constant excitation light intensity, ensuring identical detection conditions for all experimental groups and time points. Five fixed fields (upper, lower, center, left and right) were photographed per well to reduce sampling error. All images were processed uniformly in ImageJ: images were converted to 8-bit grayscale, and background signals were subtracted using the rolling ball algorithm (radius = 50 pixels). The mean gray value of the entire image was then calculated for each field. For each well, the average of the five fields was taken as the representative value. Each recombinant virus was tested in three independent wells as biological replicates. Meanwhile, to further assess the alterations in replication characteristics of recombinant viruses following exogenous gene insertion at different sites, the growth curves of stably fluorescent-expressing recombinant viruses were plotted on the basis of viral titers and genomic copy numbers, with reference to the growth curve assay described above.

### Multiple fluorescent protein expression strategies at D14AB and D19A sites

To explore the optimal construction strategy for high-titer multivalent attenuated fowl adenovirus vectors, recombinant viruses coexpressing three fluorescent proteins were constructed using the rFAdV-4 ON1 strain, which has been validated safe in 21-day-old SPF chickens. Two insertion strategies were utilized. First, 2A peptide-linked polycistronic cassettes expressing eGFP-mCherry or eGFP-mCherry-mTagBFP under a single eCMV promoter were separately integrated into optimal exogenous insertion sites. Second, an eGFP-mCherry polycistronic cassette and a mTagBFP monocistronic cassette were inserted into two selected genomic sites. Following the aforementioned screening protocol for optimal expression at each insertion site, recombinant viruses were serially passaged five times in LMH cells to preliminarily select candidates with sustained high titers and stable fluorescent protein expression. Growth curves based on viral titers and genomic copy numbers evaluated the impact of exogenous gene insertion on recombinant virus replication.

Selected high-titer recombinant viruses with stable fluorescence were further serially passaged up to passage 15. PCR was performed with primers flanking each insertion site. Target fragments from passage 10 and 15 were amplified with the aforementioned high-fidelity DNA polymerase and verified via nanopore sequencing to evaluate the genetic stability of exogenous inserts. The inserted fragment at the D14AB site was amplified with primers idD14AB F and idD14AB R, and the counterpart at the D19A site was amplified with primers idD19A F and idD19A R. All primer sequences are listed in Additional file 1. Viral titers at these passages were also determined. The maximum permissible passage was defined as the passage with intact inserted sequences, no base mutations, and titers comparable to the wild-type CH_JS_2017 strain.

For safety evaluation, 15 14-day-old SPF chickens were randomly divided into three equal groups. Two groups were intramuscularly inoculated with the maximum-passage recombinant viruses or wild-type CH_JS_2017 at 10^7.0^ TCID_50_​ per chicken. The third (mock control) group received an equal volume of DMEM/F12. The experimental observation period lasted 14 days, during which mortality in each group was recorded. At the end of the observation period, all surviving chickens were euthanized by CO_2_ asphyxiation.

### Statistical analysis

Statistical analysis was performed using GraphPad Prism 9.5.1 (GraphPad Software). A two-way analysis of variance (ANOVA) or log-rank test was applied after confirming normality (Shapiro–Wilk test) and homogeneity of variances (Levene’s test). Tukey’s post hoc test was used for all pairwise comparisons, while Dunnett’s test was applied for comparisons against a control group. Statistical significance was defined as *P < 0.05, **P < 0.01, ***P < 0.001, or ****P < 0.0001. Sample size (*n*) for each experiment is presented in figure legends.

## Results

### Effects of ORF deletions on FAdV-4 viral replication ability

**Figure 1:**
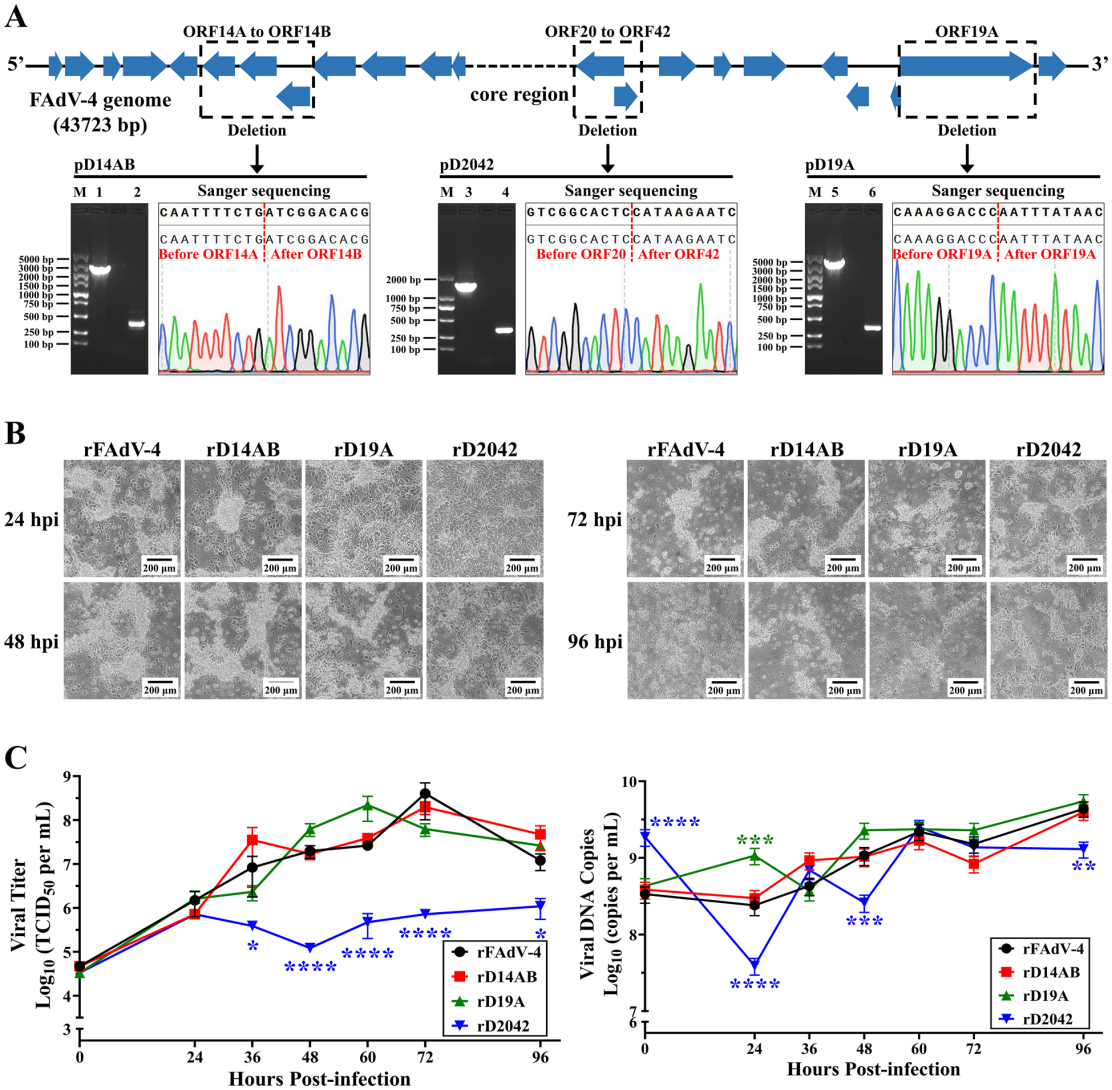
**Construction of ORF-deleted FAdV-4 strains and characterization of their growth kinetics in LMH cells**. **A** Generation of ORF-deleted FAdV-4 infectious clones pD14AB, pD19A, and pD2042. **B** CPE of LMH cells at 24 hpi, 48 hpi, 72 hpi, and 96 hpi before harvesting (MOI = 0.05). A mock control consisting of uninfected LMH cells cultured under identical conditions was included in this experiment, and no obvious CPE was observed throughout the observation period. **C** One-step growth curves of different FAdV-4 strains, which were determined by viral titers and viral DNA copy numbers, respectively. Statistical differences in data were analyzed by two-way ANOVA followed by Dunnett’s multiple comparisons test (*P < 0.05, **P < 0.01, ***P < 0.001, ****P < 0.0001 versus rFAdV-4 group). Note: all statistically significant differences are indicated in the figure. To avoid visual clutter, non-significant differences (*P* > 0.05) were omitted from the figure. Data are presented as mean ± SEM (*n* = 3).

Based on previously published studies [14, 26, 27, 32, 35], we selected ORF14A, ORF14, ORF14B, ORF20, ORF42, and ORF19A to investigate their impact on FAdV-4 replication and pathogenicity. These ORFs are in three regions in the FAdV-4 genome, and their deletion provides three potential sites for gene insertion (D14AB site, D2042 site, and D19A site). Three FAdV-4 infectious clones with deletions in different ORFs were constructed and verified by PCR and sequencing (Figure 1A). Then viruses were rescued from these infectious clones and named rD14AB, rD19A, and rD2042. After five passages on LMH cells, the growth kinetics of these recombinant viruses were evaluated.

As shown in Figure 1B, the cells inoculated with rFAdV-4 and rD14AB exhibited severe CPE at 24 hpi, while those inoculated with rD19A and rD2042 displayed milder CPE characterized by cell aggregation. Between 48 and 72 hpi, LMH cells in all groups showed pronounced CPE, including clustering and detachment. By 96 hpi, most cells inoculated with rFAdV-4, rD14AB, and rD19A had lysed, with aggregated and detached clusters disintegrating or disappearing. In contrast, cells inoculated with rD2042 still showed detached clusters, although some cells remained adherent and displayed near-normal morphology. A mock group using uninfected LMH cells cultured under identical conditions was used as the negative control, and no obvious cytopathic effects were detected at any time point.

In the one-step growth curve analysis (Figure 1C), the TCID_50_ levels of rD14AB and rD19A at various time points showed no significant differences compared with those of rFAdV-4. However, starting from 36 hpi, the viral TCID_50_ levels of rD2042 were significantly lower than those of rFAdV-4 at corresponding time points, and this difference remained significant up to 96 hpi. Within 96 h, the peak viral titers for rFAdV-4, rD14AB, and rD19A were 10^8.60^ TCID_50_/mL, 10^8.30^ TCID_50_/mL, and 10^8.34^ TCID_50_/mL, respectively. rD19A reached the peak at 60 hpi, earlier than that of rFAdV-4 (peak at 72 hpi). By contrast, the highest virus titer of rD2042 was 10^6.04^ TCID_50_/mL.

In the viral DNA copy number analysis (Figure 1C), rD19A exhibited a significantly higher viral DNA copy number than rFAdV-4 at 24 hpi, whereas the viral DNA copy number of rD2042 was significantly lower than that of rFAdV-4. From 36 to 96 hpi, the DNA copy numbers of rD14AB and rD19A showed no significant differences from those of rFAdV-4, while rD2042 exhibited significantly lower DNA copy numbers at 48 hpi and 96 hpi.

The combined deletion of ORF14A, ORF14, and ORF14B (rD14AB) or the deletion of ORF19A (rD19A) did not significantly affect the replication ability of recombinant viruses in LMH cells, whereas the combined deletion of ORF20 and ORF42 significantly impaired the virus’s in vitro replication ability, resulting in a significant decrease in viral titer compared with that of rFAdV-4.

### Effects of ORF deletions on viral pathogenicity to chickens

**Figure 2:**
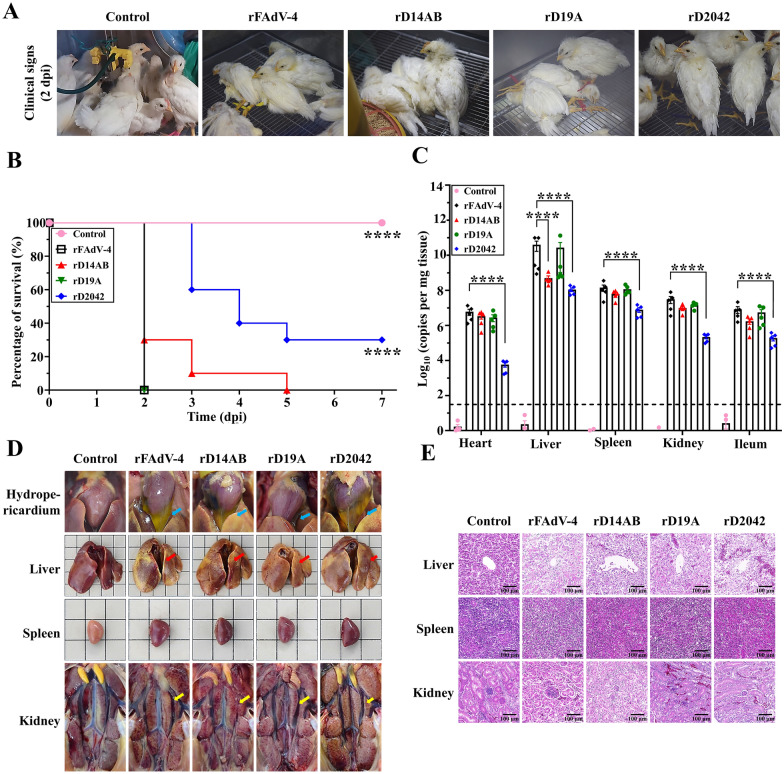
**The pathogenicity of ORF-deleted FAdV-4 strains in 21-day-old SPF chickens at the same challenge dose (3 × 10**^**6.5**^ TCID_**50**_**/bird**). **A** Clinical signs (lethargy, ruffled feathers) observed at 2 dpi. **B** Survival curves of chickens within 7 days postchallenge. **C** Viral loads in tissues at 2 dpi, data are presented as the mean ± SEM. **D** Gross lesions induced by virus infection at 2 dpi: the blue arrow indicates pericardial effusion in infected chickens; the red arrow indicates hepatic yellowish discoloration; and the yellow arrow indicates renal discoloration and swelling due to urate deposition. **E** Histopathological changes in the liver, spleen, and kidney at 2 dpi: extensive hepatocyte necrosis with disrupted hepatic cord structure; lymphocytic depletion and blurred corticomedullary boundary in spleen; extensive lysis and necrosis of renal tubular epithelial cells. The first subgroup (*n* = 10) was exclusively used for mortality analysis (panels **A**, **B**); the second subgroup (*n* = 5) was applied for early detection and excluded from mortality statistics (panels **C**–**E**). Statistical analysis: Log-rank test for survival data (**B**); two-way ANOVA followed by Dunnett’s multiple comparisons test for viral loads (**C**). Significance levels: *P < 0.05, **P < 0.01, ***P < 0.001, ****P < 0.0001 versus control group (**B**) or rFAdV-4 group (**C**). The threshold line added in Figure 2C was calculated as 30.8 copies/mg from the lower limit of quantification (LLOQ) of the standard curve (100 copies/μL) and the maximum tissue weight of the negative control group (430 mg), as described in Materials and Methods. Data below this threshold line have no actual quantitative significance, do not represent true viral copy numbers, and are presented for theoretical reference only.

Furthermore, to investigate whether the in vivo replication capacity and pathogenicity of the previously constructed recombinant viruses (with deletions of different ORFs) were affected by the ORF deletions, we inoculated 21-day-old SPF chickens with the aforementioned recombinant viruses and wild-type FAdV-4 (rFAdV-4) at the same challenge dose (3 × 10^6.5^ TCID_50_/bird). At 1 dpi, chickens in the rFAdV-4, rD14AB, rD19A, and rD2042 groups exhibited lethargy, ruffled feathers (Figure 2A), as well as huddling and crouching behaviors. At 2 dpi, the rFAdV-4, rD14AB, and rD19A groups showed high mortality, whereas no significant mortality was observed in the rD2042 group until 3 dpi. By the end of the observation period (7 dpi), the rFAdV-4, rD14AB, and rD19A groups had a 100% mortality rate. In contrast, the rD2042 group had a morbidity rate of 100% (all chickens showed clinical signs including lethargy and ruffled feathers) but a mortality rate of 70% (Figure 2B). Both morbidity and mortality were calculated solely based on the first subgroup (*n* = 10), excluding the additional five birds per group.

Measurement of viral load in tissues at 2 dpi showed that the liver had a higher viral load than other tissues, with viral DNA copies up to 10^8^ copies/mg in all infected groups (Figure 2C). Only the liver viral load of the rD14AB group was significantly reduced relative to the rFAdV-4 group, while viral loads in all other tissues and in the rD19A group remained comparable to those of the wild-type strain. Notably, viral loads in all collected tissues were significantly lower in the rD2042 group than in the wild-type group. Consistent with the in vitro replication phenotypes in LMH cells (Figure 1C), dual deletion of ORF20 and ORF42 remarkably impaired the in vivo replication of rD2042 in SPF chickens. Nevertheless, sporadic background amplification signals (Ct values 36.41–38.47) were detected in several organs of negative control chickens, which were deemed nonspecific and did not represent genuine viral replication per our predefined assay threshold.

At necropsy (Figure 2D), chickens from the rFAdV-4, rD14AB, rD19A, and rD2042 groups exhibited marked pericardial effusion that was clear and pale yellow (indicated by blue arrows). In chickens from all infected groups, the livers exhibited yellowish discoloration (indicated by red arrows); the spleens were enlarged and purplish, and the kidneys were swollen with surface wrinkling and discoloration (indicated by yellow arrows). Histopathological changes were observed in organs under microscopic examination (Figure 2E). In the liver, hepatocytes from the rFAdV-4, rD14AB, rD19A, and rD2042 groups exhibited extensive lysis, with cytoplasmic staining becoming pale, hepatic cord structure disappearing, and red blood cell infiltration observed in the liver stroma. In the spleen, tissues from all infected groups exhibited lymphocytic depletion, loss of splenic structure, and indistinct boundaries between the cortex and medulla. In the kidneys, tubular epithelial cells from the rFAdV-4, rD14AB, rD19A, and rD2042 groups exhibited extensive lysis and necrosis. This indicates that although combined deletion of ORF20 and ORF42 impairs the in vivo replication capacity of rD2042, this mutant virus can still induce typical pathological lesions of FAdV-4.

In summary, deletion of ORF14A/14/14B only restricted viral replication in the liver without attenuating viral pathogenicity, as reflected by unchanged 100% mortality. Knockout of ORF19A exerted no obvious impacts on the in vivo replication or virulence of rD19A. In contrast, combined deletion of ORF20 and ORF42 strongly suppressed viral proliferation in major target organs and effectively reduced the mortality of infected chickens to 70%.

### Transcription termination sequences play an important role in recombinant virus passage and foreign gene expression

**Figure 3:**
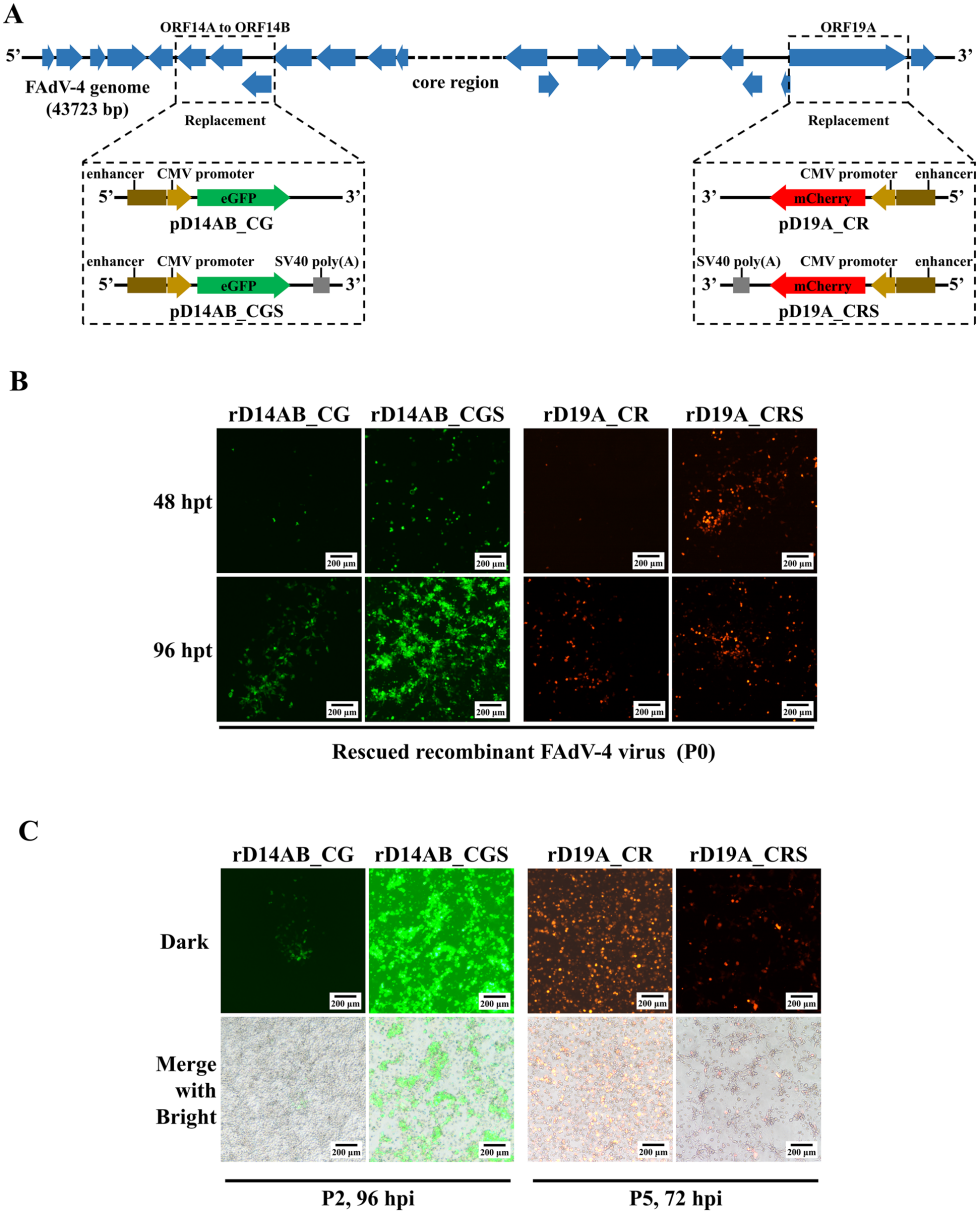
**Construction of recombinant FAdV-4 viruses expressing single-fluorescent protein at D14AB or D19A sites, respectively.** **A** Schematic diagram illustrating the construction of recombinant FAdV-4 strains. **B** Rescue of single-fluorescent-protein recombinant FAdV-4 strains. At 48 h and 96 h post-transfection with the infectious clone plasmids, the expected color of fluorescence was observable for all single-fluorescent recombinant viruses under an inverted fluorescence microscope. **C** Passage stability of recombinant FAdV-4 strains with or without the SV40 poly(A) terminator (Only data on the passage with the best fluorescent expression effect and the corresponding observation time in each recombinant virus were presented).

Given their minimal impact on viral replication (in vitro and in vivo), we selected D14AB and D19A sites to assess exogenous gene expression potential. Considering that transcription termination sequences may disrupt the transcription of native genes, fluorescent protein expression cassettes were designed with or without such a terminator. In building recombinant viral exogenous gene cassettes, we minimized the sequence length of transcriptional elements. Therefore, we chose the SV40 poly(A) signal—compact, well-annotated, and efficient in diverse eukaryotic systems—for enhancing mRNA stability and protein yield. Compared with longer poly(A) signals (e.g., bovine growth hormone (bGH)-derived), SV40 poly(A) is more practical for vectors with limited insertion length [36] (Figure 3A). Viable viruses were successfully rescued from the recombinant infectious clones. Both green and red fluorescence were observed under the fluorescence microscope (Figure 3B).

For the D14AB site, at an identical MOI of 0.05, the terminator-free rD14AB_CG showed markedly delayed CPE with only faint fluorescence, whereas the SV40 poly(A)-carrying rD14AB_CGS developed typical cytopathic changes (Figure 3C). At the D19A site, rD19A_CRS with the terminator exhibited accelerated syncytia formation and CPE progression. Enhanced viral replication and release narrowed the fluorescent protein expression window, resulting in reduced fluorescence intensity (Figure 3C).

To verify these results, P2 viruses were inoculated into LMH cells at MOI 0.05, harvested at 96 hpi, and P3 titers were determined. At passage 3, rD14AB_CG lost obvious CPE and fluorescent expression, while rD14AB_CGS maintained a stable titer of 10^8.00^ TCID_50_​/mL. Both D19A recombinant viruses retained normal fluorescence; however, rD19A_CRS yielded a 0.5-log higher titer (10^9.50^ TCID_50_​/mL) and faster CPE than rD19A_CR (10^9.00 ^TCID_50_​/mL).

In summary, the D14AB-inserted expression cassette required the SV40 poly(A) terminator to sustain viral genetic stability and continuous fluorescence expression. Mechanistically, the D14AB site may be adjacent to genes associated with viral replication complex assembly. Expression cassettes lacking the SV40 poly(A) signal may trigger transcriptional read-through and generate abundant antisense transcripts relative to the aforementioned genes, thereby silencing their expression. In contrast, D19A site is distant from essential transcription units, so the SV40 terminator barely affects viral fitness, allowing stable passage regardless of terminator presence. However, when terminator-deficient expression cassettes are inserted into the D19A site, the strong transcriptional activity of the eCMV promoter may produce aberrantly long read-through transcripts and interfere with the alternative splicing of ORFs in the same transcriptional orientation. Accordingly, the delayed replication of rD19A_CR is probably attributed to interference with the expression of viral replication-associated ORFs downstream of the exogenous cassette, thereby allowing sufficient time for protein expression at an identical MOI.

### Growth kinetics of single-fluorescent recombinant FAdV-4 strains and levels of fluorescent protein

**Figure 4:**
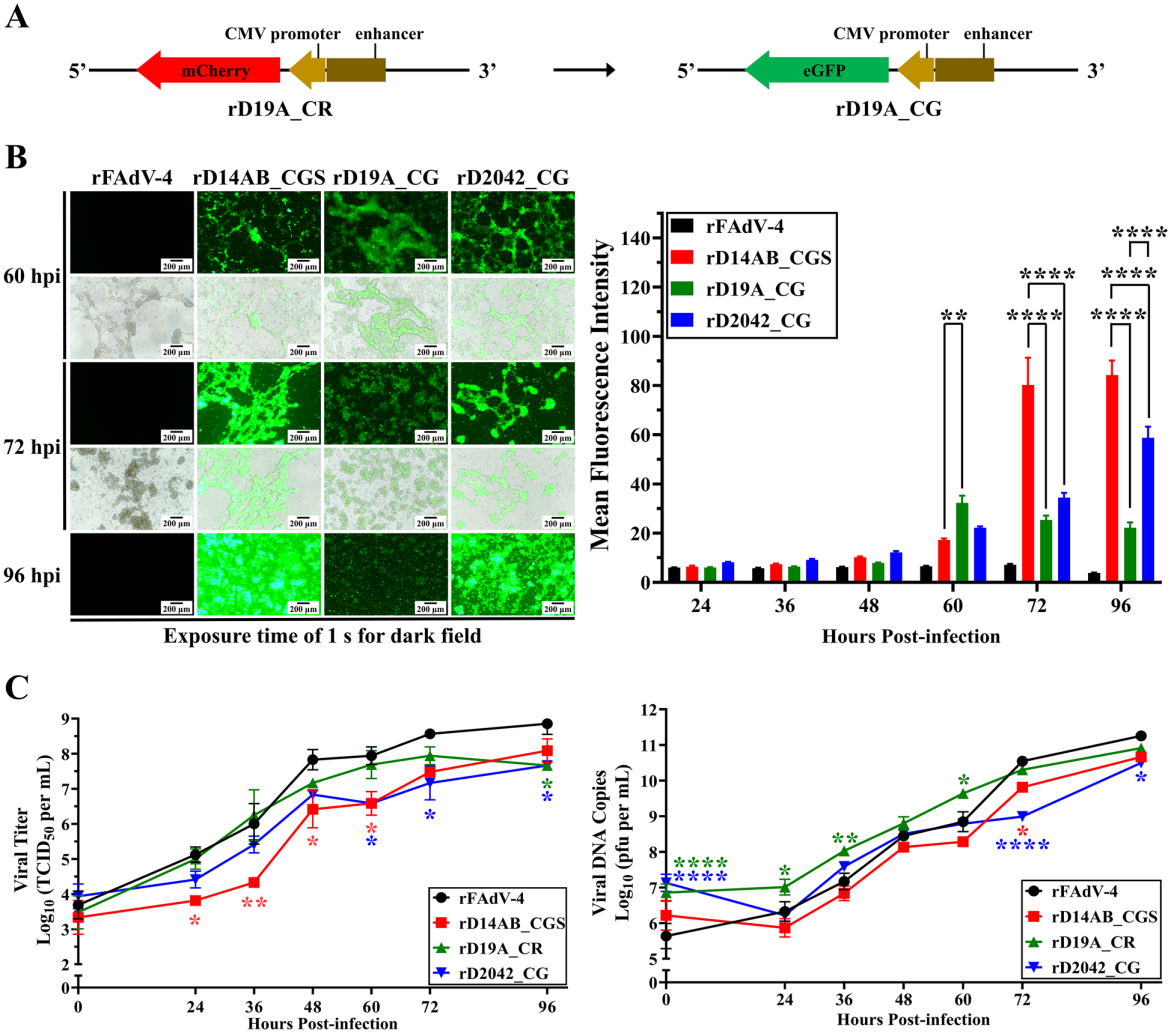
**Levels of eGFP expressed by recombinant FAdV-4 strains and growth kinetics of these viruses.** **A** Replacing the mCherry codon sequence of rD19A_CR with eGFP coding sequence. **B** Levels of eGFP expressed by rD14AB_CG and rD19A_CG. Mean fluorescence intensity was measured using Image J. **C** One-step growth curves of different FAdV-4 strains determined by viral titer and viral DNA copy numbers, respectively. All data in this figure are presented as the mean ± SEM (*n* = 3). Statistical differences in data were analyzed by two-way ANOVA followed by Dunnett’s multiple comparisons test (*P < 0.05, **P < 0.01, ***P < 0.001, ****P < 0.0001 versus the rFAdV-4 group).

With the optimal expression-cassette design for each locus established (i.e., with or without the SV40 terminator), we sought to identify the best site for foreign-protein expression by comparing both the expression level of a single fluorescent protein and the resulting impact on viral replication in vitro. To enable a direct comparison of expression levels, we constructed recombinant viruses carrying an eGFP cassette at the D14AB (rD14AB_CGS) and D19A (rD19A_CG) sites (Figure 4A). For a broader context, we also included the D2042 site, despite its known impact on replication, to fully assess its potential (Additional file 3).

We first monitored eGFP expression over time (Figure 4B). While initial fluorescence (before 48 hpi) was low and comparable across all viruses, intensity increased rapidly thereafter. Notably, the fluorescence intensity of rD19A_CG peaked at 60 hpi and subsequently declined, whereas the signals from rD14AB_CGS continued to increase. By 96 hpi, rD14AB_CGS exhibited the highest eGFP expression levels, significantly surpassing rD19A_CG.

We next assessed the impact of the insertions on viral replication through one-step growth curves and viral DNA copy number analysis (Figure 4C). The titers of rD14AB_CGS and rD19A_CG were generally lower than those of the wild-type rFAdV-4, confirming that the expression cassettes impose a fitness cost. Among the recombinants, rD19A_CG titers were closest to the wild type, showing no significant difference (*P* > 0.05). In contrast, the viral titer of rD14AB_CGS was approximately one order of magnitude lower than that of wild‑type rFAdV‑4 strain at 72 hpi. Specifically, the titer reached 10^7.45^ TCID_50_​/mL, while that of rFAdV‑4 was 10^8.56^ TCID_50_​/mL.

Consistent with our earlier cassette design, the eGFP cassette at the D2042 site also could not contain an SV40 poly(A) terminator (Additional file 3). Notably, insertion of the eGFP expression cassette lacking the SV40 poly(A) terminator at the D2042 locus partially restored the replication capacity of rD2042_CG in LMH cells. At an identical MOI of 0.05, the titer of rD2042_CG reached 10^7.66^ TCID_50​_/mL at 96 hpi (Additional file 3), whereas the dual ORF20/ORF42-deleted rD2042 only yielded a titer of 10^6.04^ TCID_50_​/mL (Figure 1). Nevertheless, compared with wild-type FAdV-4, its genomic DNA replication was still significantly impaired at 72 hpi (*P* < 0.01) or 96 hpi (*P* < 0.05) (Additional file 3). We therefore speculate that, although the proteins encoded by ORF20 and ORF42 are nonessential for FAdV-4, dual deletion of these two open reading frames may interfere with the expression of genes related to viral genome replication, thereby severely impairing viral replication competence. After insertion of exogenous cassettes without the SV40 poly(A) terminator, the eCMV promoter may drive the upregulation of downstream replication‑regulatory genes, which consequently partially restores the replication capacity of the recombinant virus. Considering the impact of ORF20 and ORF42 deletion on the in vivo replication capacity of recombinant viruses (Figure 2), as well as the significant decrease in genomic DNA copy number observed at 24 h postinfection (hpi) in LMH cells (Additional file 3), we still did not select the D2042 site as a subsequent insertion site.

The results show that, at the same MOI, the D14AB site produces the highest single-fluorescent-protein expression but exerts a relatively greater negative effect on in vitro replication of the recombinant viruses. By contrast, the D19A site minimizes impact on recombinant-virus replication while still providing adequate foreign-protein expression. Therefore, both sites are suitable for foreign-protein insertion, offering different trade-offs between expression level and replication fitness.

### Differential insertion capacities of the D14AB and D19A sites for exogenous gene expression

**Figure 5:**
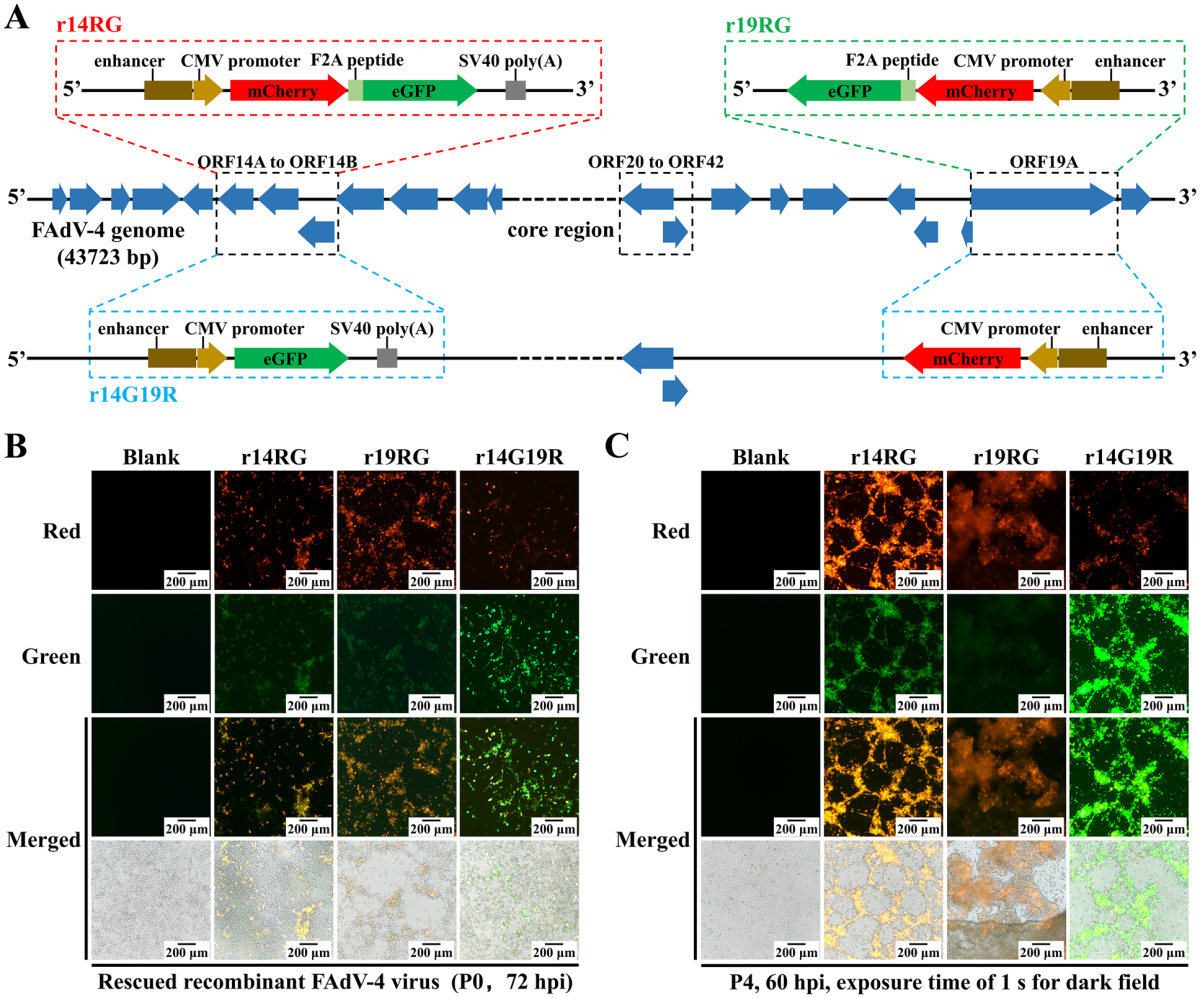
**Construction of recombinant FAdV-4 strains expressing double-fluorescent proteins.** **A** Schematic diagram illustrating the construction of recombinant FAdV-4 strains. **B** Rescue of double-fluorescent-protein recombinant FAdV-4 strains. The data shown in the figure demonstrate the expression of fluorescent proteins in each infectious clone of recombinant viruses at 72 h post-transfection (i.e., passage 0). **C** Passage stability of double-fluorescent recombinant FAdV-4 strains. The data shown in the figure demonstrate the expression of fluorescent proteins in each recombinant virus 60 h after infection, following four serial passages in LMH cells.

To further explore the rational construction strategy of multivalent poultry vaccines, the capacity of the D14AB and D19A sites to accommodate exogenous genes was assessed by utilizing dual fluorescent protein expression cassettes in this section. Meanwhile, a recombinant virus that expressed eGFP at the D14AB site and mCherry at the D19A site—each via an independent expression cassette—was constructed (Figure 5A).

As shown in Figure 5B, in the recombinant FAdV-4 strains r14RG and r19RG, where the coding sequences (CDS) of mCherry and eGFP were located within the same expression cassette and linked by the foot-and-mouth disease virus (FMDV) 2A (F2A) self-cleaving peptide, the expression level of mCherry (positioned upstream of the 2A peptide) was higher than that of eGFP (positioned downstream), and red fluorescence colocalized with green fluorescence. When the CDS of mCherry and eGFP were placed in two separate expression cassettes in the recombinant virus r14G19R, the expression level of eGFP at the D14AB site was higher than that of mCherry at the D19A site, and only partial colocalization of red and green fluorescence was observed.

In Figure 5C, the results of continuous passaging of the recombinant viruses indicate that r14RG could be stably passaged and could maintain expression of both mCherry and eGFP. Although r19RG could also be passaged and still coexpress mCherry and eGFP, the expression level of eGFP decreased gradually over successive passages.

These results demonstrate that the D14AB site can support the insertion of longer fragments (i.e., expression cassettes containing two fluorescent proteins, which are longer in length), whereas the D19A site has a more limited capacity and can only accommodate shorter fragments (i.e., expression cassettes containing a single fluorescent protein). Moreover, these two sites can independently support the expression of exogenous proteins within the same FAdV-4 genome when using separate expression cassettes.

### Exploration of construction strategies for multivalent attenuated vaccine vectors based on fluorescent protein expression cassettes

To construct a high-titer multivalent attenuated vaccine vector expressing multiple exogenous proteins, we applied our prior findings to explore exogenous protein expression strategies for the previously established fully attenuated strain rON1 [33].

**Figure 6:**
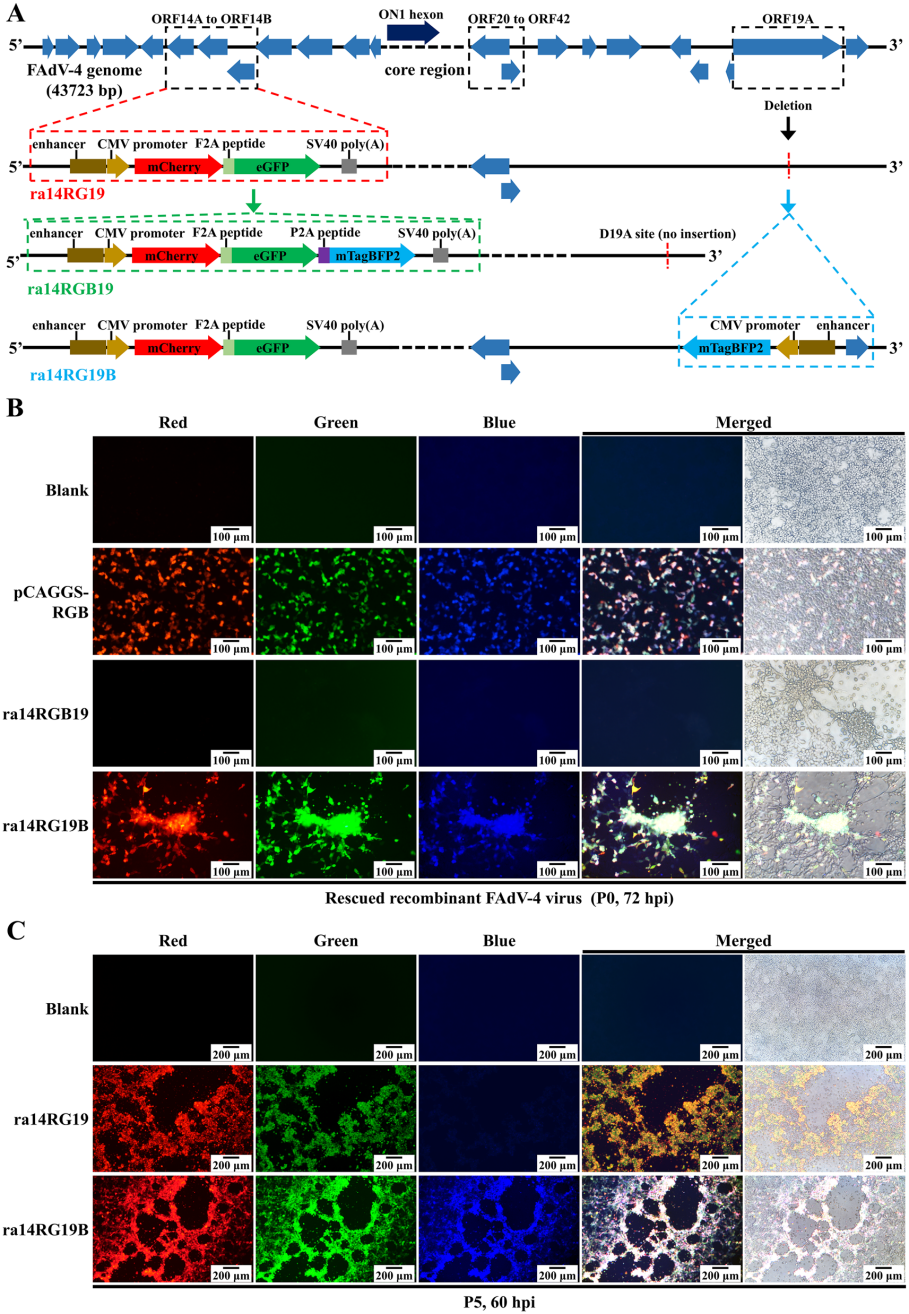
**Construction of recombinant FAdV-4 strains expressing triple-fluorescent proteins.** **A** Schematic diagram illustrating the construction of recombinant FAdV-4 strains. F2A: the foot-and-mouth disease virus 2A peptide; P2A: the porcine teschovirus-1 2A peptide. **B** Rescue of double and triple-fluorescent-protein recombinant FAdV-4 strains. pCAGGS-RGB refers to the construct generated in this study, where a tri-color fluorescent protein expression cassette was inserted into the expression frame of the eukaryotic expression vector pCAGGS. This construct was used to verify that the designed tri-color fluorescent protein expression cassette could be normally expressed in LMH cells. **C** Passage stability of double and triple-fluorescent recombinant FAdV-4 strains. The data shown in the figure represent the luminescence of tri-color fluorescent proteins at 60 hpi of LMH cells by the 5th passage of recombinant FAdV-4—where the recombinant FAdV-4 expresses tri-color fluorescent proteins and had undergone 5 consecutive passages in LMH cells prior to this infection.

First, we previously found ORF19A knockout promoted viral genomic DNA replication to some extent. To mitigate titer reduction in recombinant viruses due to exogenous gene insertion, all three-fluorescent protein-expressing recombinant attenuated viruses constructed here underwent ORF19A knockout—either via direct deletion or insertion of a blue fluorescent protein (mTagBFP2) cassette at this site (Figure 6A).

Second, to further define the maximum exogenous gene insertion length for the attenuated vector, we designed two recombinant viruses expressing three fluorescent proteins: one with three fluorescent protein cassettes (linked via self-cleaving 2A peptides) at D14AB, and the other with two cassettes at D14AB and one at D19A (Figure 6A).

In the rescue of constructed recombinant viruses, only ra14RG19 and ra14RG19B could be rescued and could express fluorescent proteins (Figure 6B; ra14RG19 data not shown). Although ra14RGB19 could be rescued, no fluorescence could be observed under a fluorescence microscope (Figure 6B). Notably, the two successfully rescued strains (ra14RG19 and ra14RG19B) maintained high and stable expression of the inserted fluorescent genes after five consecutive passages in LMH cells (Figure 6C). To rule out the possibility of design defects in the triple fluorescent protein cassette itself, we verified its expression in the pCAGGS plasmid, which showed successful expression, confirming that the cassette had no structural or design defects (Figure 6B).

Since the triple fluorescent protein cassette itself was confirmed to be functional, we further clarified the mechanism underlying the defective fluorescence phenotype of ra14RGB19 by determining viral titers. Second-generation viral stocks of ra14RGB19 and ra14RG19 were inoculated into LMH cells at an identical MOI of 0.05. Viral supernatants were collected at 96 hpi for titer determination. The results demonstrated that ra14RGB19 was still capable of inducing CPE in LMH cells. Nevertheless, the titer of passage 3 ra14RG19 reached 10^8.67^ TCID_50_​/mL, while that of ra14RGB19 was only 10^5.50^ TCID_50​_/mL, representing a difference of approximately three orders of magnitude.

In conclusion, combined with the above results, insertion of an excessively long exogenous fragment exceeds the maximum packaging and insertion capacity of the D14AB site, causing severe impairment of viral replication. The resultant reduction in viral genome copy numbers leads to insufficient transcriptional templates, which ultimately abolishes the expression of the triple fluorescent protein cassette in ra14RGB19.

### Growth kinetics of multivalent attenuated vaccine vectors expressing fluorescent protein

**Figure 7:**
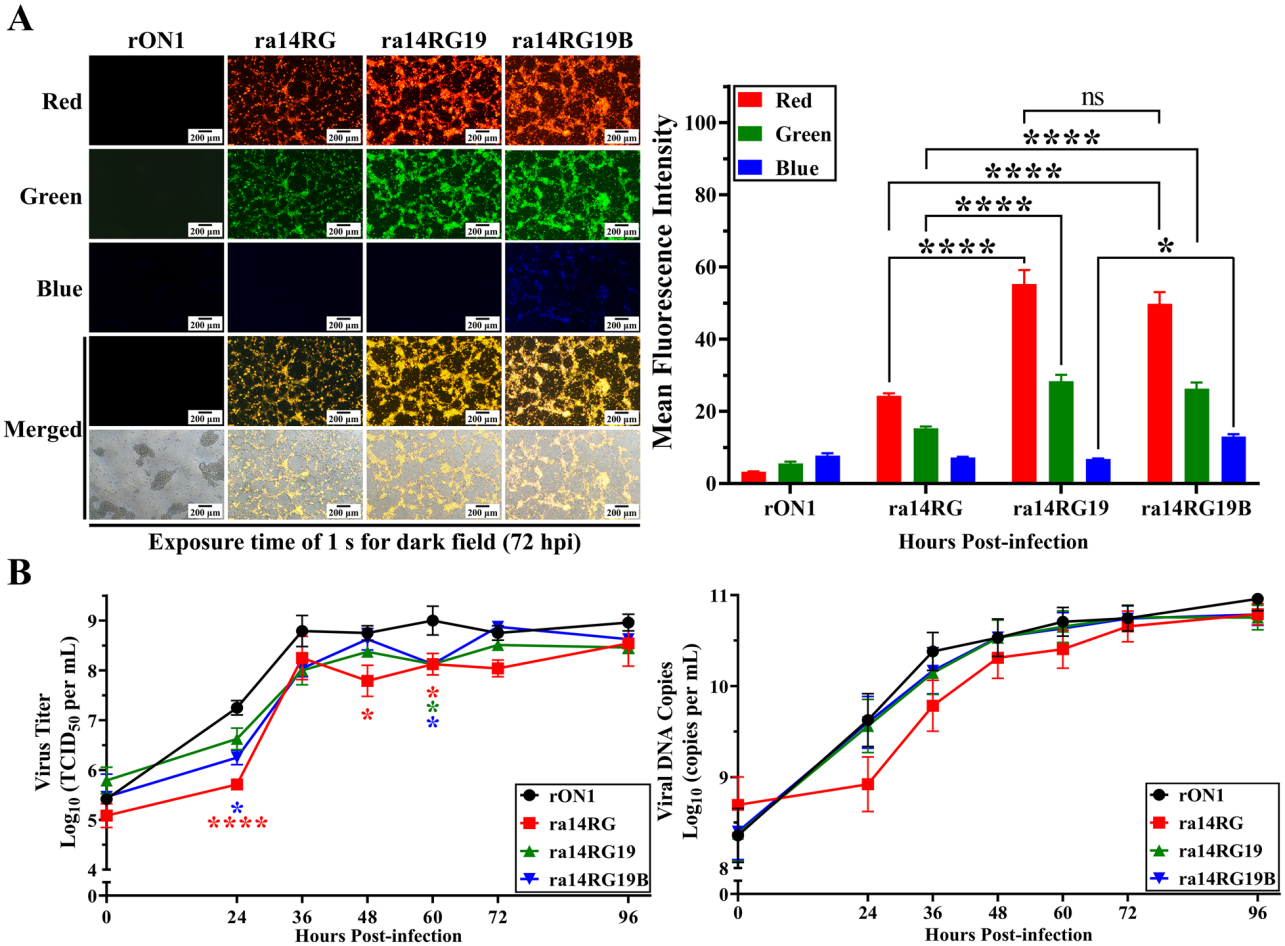
**Levels of fluorescent proteins expressed by recombinant virus strains and growth kinetics of these viruses.** **A** Levels of fluorescent proteins expressed by recombinant FAdV-4 strains. ra14RG: with ORF19A; ra14RG19 and ra14RG19B: ORF19A-deleted. Mean fluorescence intensity was measured using Image J. **B** One-step growth curves of ra14RG, ra14RG19, and ra14RG19B, which were determined by viral titers and viral DNA copy numbers, respectively. Statistical differences in data were analyzed by two-way ANOVA followed by Dunnett’s multiple comparisons test (*P < 0.05, **P < 0.01, ***P < 0.001, ****P < 0.0001 versus the rFAdV-4 group). Note: all statistically significant differences are indicated in the figure. To avoid visual clutter, only the key non-significant (ns) comparison most likely to cause reader confusion is explicitly labeled (*P* > 0.05). Data are presented as mean ± SEM (*n* = 3).

To further evaluate the construction strategies for the multivalent attenuated vaccine strain, we inoculated LMH cells with fifth-passage viruses at the same MOI and assessed for foreign-protein expression, namely mean fluorescence intensity (MFI**)**, as well as in vitro replication (one-step growth curves). At 72 hpi (Figure 7A), mCherry and eGFP MFIs from ra14RG19 and ra14RG19B (both ORF19A-deleted FAdV-4 derivatives) were significantly higher than those from ra14RG. In ra14RG19B, insertion of mTagBFP2 at the D19A site modestly reduced mCherry and eGFP expression from the D14AB site; although MFIs for ra14RG19B trended lower than ra14RG19, the difference was not statistically significant. One-step growth curves (Figure 7B) showed that ra14RG19 and ra14RG19B replicated more efficiently than ra14RG. Peak titers were: rON1, 10^8.99^ TCID_50_/mL (60 hpi); ra14RG, 10^8.62^ TCID_50_/mL (96 hpi); ra14RG19, 10^8.50^ TCID_50_/mL (72 hpi); and ra14RG19B, 10^8.87^ TCID_50_/mL (72 hpi). Together, these data indicate that the foreign-protein expression strategies identified in wild-type FAdV-4 are compatible with the attenuated rON1 backbone: the D14AB locus can support simultaneous expression of two different foreign proteins, and deletion or replacement of ORF19A (for example with an mTagBFP2 cassette) can mitigate the replication inhibition associated with D14AB expression, enabling attenuated vectors to reach titers comparable to rON1. Although replacing ORF19A reduces expression from D14AB, it increases the number of foreign genes the vector can carry, thereby broadening its potential as a multivalent vaccine platform.

For a systematic overview, the genotypes, insertion sites, and peak titers of all recombinant viruses constructed in this study are summarized in Table 1. Among these, ra14RG19B, which uniquely carries three distinct fluorescent reporters and achieved the highest peak titer among all recombinant viruses expressing foreign proteins, was selected for in-depth evaluation of genetic stability and in vivo safety. The virus (ra14RG19B) was serially passaged for 15 consecutive generations in LMH cells. Inserted fragments at the D14AB and D19A sites from passages 10 to 15 were analyzed by PCR, and nanopore sequencing was further performed to verify exogenous sequences at passages 10 and 15. The results demonstrated that the D14AB site exhibited favorable genetic stability. The inserted fragment remained intact without base mutations throughout 15 serial passages (Additional file 4A; detailed sequencing data are provided in Additional file 5). In contrast, exogenous sequences at the D19A site began to be lost after passage 13 (Additional file 4B; Additional file 5). Sequencing assembly identified two distinct sequence patterns at the D19A site in passage 10 viruses. Approximately 16% of fragments lacked the mTagBFP2 ORF while retaining partial elements of the exogenous expression cassette, indicating early outgrowth of deletion mutants. These results demonstrated that foreign gene deletion at the D19A site of ra14RG19B first appeared at passage 10, and this recombinant virus only maintained favorable genetic stability within the first 10 passages.

**Table 1:** **Summary of key information for all FAdV-4 strains rescued in this study**

Strain	Backbone	Genetic modification	Peak titer (log_10_​TCID_50_​/mL)
rFAdV-4	CH_JS_2017	Wild-type, no genetic modification	8.60
rD14AB	rFAdV-4	ORF14A/14/14B deletion, no exogenous gene	8.30
rD19A	rFAdV-4	ORF19A deletion, no exogenous gene	8.34
rD2042	rFAdV-4	ORF20/42 deletion, no exogenous gene	6.04
rD14AB_CG	rFAdV-4	eGFP cassette inserted at D14AB, without SV40 poly(A)	–^1^
rD14AB_CGS	rFAdV-4	eGFP cassette inserted at D14AB, with SV40 poly(A)	8.06
rD19A_CR	rFAdV-4	mCherry cassette inserted at D19A, without SV40 poly(A)	8.00
rD19A_CRS	rFAdV-4	mCherry cassette inserted at D19A, with SV40 poly(A)	8.50
rD19A_CG	rFAdV-4	eGFP cassette inserted at D19A, without SV40 poly(A)	8.56
rD2042_CG	rFAdV-4	eGFP cassette inserted at D2042, without SV40 poly(A)	7.66
rD2042_CGS	rFAdV-4	eGFP cassette inserted at D2042, with SV40 poly(A)	–^1^
r14RG	rFAdV-4	mCherry-F2A-eGFP cassette inserted at D14AB, with SV40 poly(A)	–^1^
r19RG	rFAdV-4	mCherry-F2A-eGFP cassette inserted at D19A, without SV40 poly(A)	–^1^
r14G19R	rFAdV-4	eGFP cassette inserted at D14AB, without SV40 poly(A); mCherry cassette inserted at D19A, without SV40 poly(A)	–^1^
rON1	CH_JS_2017	*Hexon* replaced with that of attenuated ON1 strain, no exogenous gene	8.99
ra14RG	rON1	D14AB inserted mCherry & eGFP, with SV40 poly(A)	8.62
ra14RG19	rON1	ORF19A deletion + mCherry-F2A-eGFP cassette inserted at D14AB, with SV40 poly(A)	8.67
**ra14RG19B**^**2**^	**rON1**^**2**^	mCherry-F2A-eGFP cassette inserted at D14AB, with SV40 poly(A); mTagBFP2 cassette inserted at D19A, without SV40 poly(A)	**9.00 (p10)**^**2**^
ra14RGB19	rON1	ORF19A deletion + mCherry-F2A-eGFP-P2A-mTagBFP2 cassette inserted at D14AB, with SV40 poly(A)	5.50

^1^—indicates titer undetermined or viral strain unable to stably passage

^2^Bold indicates strain ra14RG19B, which was selected for subsequent genetic stability and safety evaluations

Meanwhile, to monitor alterations in viral growth properties during continuous passaging, we determined the titers of passage-10 and passage-15 ra14RG19B. The titer of passage-10 ra14RG19B reached 10^9.00^ TCID_50​_/mL, which was comparable to the wild-type FAdV-4 strain CH_JS_2017 (10^9.20^ TCID_50_​/mL). However, the titer declined sharply to 10^7.33^ TCID_50_​/mL at passage 15. Collectively, these data confirm that ra14RG19B possesses stable genetic traits and growth kinetics similar to the wild-type strain within 10 passages.

To assess the in vivo safety of ra14RG19B as a live attenuated vaccine candidate, 14-day-old SPF chickens were inoculated with passage-10 ra14RG19B or wild-type CH_JS_2017 at a dose of 10^7.00^ TCID_50​_ per bird, with five chickens in each group. During the 14-day observation period, all chickens in the CH_JS_2017-infected group died as early as 4 days postinoculation, resulting in a 100% mortality rate. In comparison, no mortality or obvious clinical abnormalities were observed in the ra14RG19B group, with health status consistent with the mock control group (Additional file 6). These results verify the excellent safety of ra14RG19B in young SPF chickens.

Consistent with our previous finding that deletion or replacement of ORF19A mitigates D14AB-associated replication inhibition, we successfully constructed a novel multivalent fowl adenovirus live attenuated vaccine candidate via systematic experimental validation. Collectively, these results demonstrate that ra14RG19B meets key criteria for a multivalent live attenuated vaccine candidate: stable foreign-gene expression and genetic integrity over 10 passages, replication kinetics comparable to wild-type FAdV-4, and a favorable safety profile in 14-day-old SPF chickens.

## Discussion

FAdV-4 can infect various poultry, making it a promising candidate for development into a recombinant live viral vector. Unlike human adenovirus type 5 (HAdV-5), which contains well-established nonessential regions such as E1 and E3 for vector engineering, FAdV-4 does not seem to possess a comparably large, continuous nonessential genomic segment. Instead, its transcriptional organization is more dispersed, with multiple promoters contributing to gene expression across the genome [32]. Moreover, the biological functions of proteins encoded by FAdV-4 nonessential genes remain largely uncharacterized, and most existing studies have been limited to analyzing the impacts of deleting individual nonessential ORFs on viral replication. In addition, the large genome size of FAdV-4 (43–45 kb) complicates efficient genomic modification, further restricting investigations into the maximum exogenous gene insertion capacity of knockout sites. Notably, even with insertion of a single fluorescent protein expression cassette, the titer of recombinant FAdV-4 decreases by at least one order of magnitude relative to the wild-type strain [14, 27]. Insertional modification of exogenous genes within the essential Fiber-2 region exerts an even more drastic effect on viral replication, reducing recombinant virus titers by roughly three orders of magnitude compared with wild-type FAdV-4 [16].

**Figure 8:**
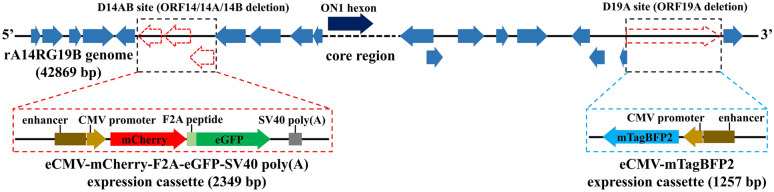
**Schematic of the dual-expression recombinant FAdV-4 vector ra14RG19B.** This schematic illustrates the full-length genome (42,869 bp) of the recombinant virus rA14RG19B, which was constructed on the basis of the rON1 backbone by inserting two foreign gene expression cassettes into the D14AB (ORF14/14A/14B deletion) and D19A (ORF19A deletion) sites, respectively. Specifically, the eCMV-mCherry-F2A-eGFP-SV40 poly(A) cassette (2349 bp) was inserted at the D14AB site, while the eCMV-mTagBFP2 cassette (1257 bp) was inserted at the D19A site.

These challenges prompted us to explore the feasibility of combinatorial deletion of nonessential ORFs, with the goal of generating recombinant viruses that coexpress multiple exogenous genes while retaining high replication titers. Accordingly, building on our prior success in efficient FAdV-4 genome modification using λ-Red homologous recombination [33], we optimized the length of homology arms to 200–300 bp in this study to further improve genome editing efficiency. Using this optimized platform, we confirmed that the D14AB site (combinatorial deletion of ORF14/14A/14B, maximum insertion capacity: 2,349 bp) and the D19A site (deletion of ORF19A, maximum insertion capacity: 1,257 bp) represent ideal insertion sites that support robust exogenous gene expression with only mild impairment of viral replication. In contrast, previous single-site FAdV-4 vectors are typically limited to < 2 kb of foreign sequence and exhibit a 1–2 log reduction in viral titer compared with wild-type virus [14, 16, 27]. Our dual-site strategy, by dividing the 3.6-kb total expression cassette across two optimized sites (Figure 8), overcomes these limitations. The final dual-site recombinant virus ra14RG19B achieved stable multigene expression while maintaining a replication capacity comparable to that of the wild-type strain.

Interestingly, we found that although ORF20 and ORF42 are nonessential genes for FAdV-4 [14, 27], combinatorial deletion of both genes drastically impaired the replication of the recombinant strain rD2042 in vitro and in vivo. Based on the well-annotated transcriptional map of the prevalent Chinese FAdV-4 strain by Lu et al. [32], we found that a reverse promoter (nt 35,495) lies between ORF20 and ORF42, which controls a transcription unit encompassing ORF22, ORF20A, and ORF20 (Additional file 7A). ORF22 deletion severely compromises viral replication [27]. We therefore infer that combinatorial deletion of ORF20 and ORF42 disrupted this critical transcription unit, thereby potentially restricting ORF22 expression. In contrast, transcription of genes controlled by the adjacent forward promoter (nt 37,061) remained unaffected [32]. Thus, loss of functional ORF22 is likely a major cause of the defective replication in rD2042. This indicates that FAdV-4 genome transcription is governed by multiple promoters, making insertion-site design far more complex than for human adenoviruses. For combinatorial ORF deletion, the potential impact on viral transcription units must be considered, in addition to the functions of the ORF-encoded proteins themselves. As noted in Lu’s transcriptome map, the viral late forward promoter (9275 nt) also drives low-abundance transcription of ORF4 [27, 37] (a gene possibly linked to FAdV-4 replication efficiency), which lies downstream of the D2042 site (Additional file 7A). In this study, forward insertion of an eGFP expression cassette without the SV40 poly(A) termination signal at the D2042 site partially restored viral replication, whereas the cassette carrying SV40 poly(A) rendered the recombinant virus incapable of regular passage. As a strong eukaryotic transcriptional terminator, SV40 poly(A) in the same orientation as the late forward promoter likely blocked low-abundance ORF4 transcription, further inhibiting viral DNA replication. In the absence of this terminator, ORF4 transcription was preserved; furthermore, transcriptional extension driven by the eCMV promoter may have enhanced ORF4 expression, thereby boosting viral replication. Furthermore, the strong eCMV-driven transcriptional read-through effect may also concurrently upregulate the expression of the replication-associated GAM1 gene (Additional file 7B) [27]. If the read‑through simultaneously upregulates both ORF4 and GAM1, their combined effect could additively or synergistically enhance viral replication. Direct transcript or protein measurements will be needed to test this hypothesis. Notably, such divergent phenotypic effects driven by identical eCMV cassettes are determined by the distinct site transcriptional context of each insertion site, including adjacent promoter orientation, overlapping transcription unit arrangement, and the functional properties of downstream viral genes.

These findings provide key insights into the site-dependent effects of the SV40 poly(A) termination signal. Although deletion of the D14AB and D19A regions did not disrupt local or adjacent transcription units, the combined effects of eCMV-driven transcriptional extension and SV40 poly(A)-mediated termination from inserted exogenous cassettes may alter native FAdV-4 transcription. The D14AB site lies immediately upstream of the ORF encoding fowl adenovirus replication complex‑associated proteins, which is regulated by the reverse 17,972‑nt promoter (Additional file 7B) [32]. Forward insertion of the exogenous expression cassette lacking the SV40 poly(A) signal enables robust eCMV‑driven transcription, producing abundant antisense transcripts opposite to the transcription orientation of this ORF. This process may silence the expression of replication complex‑related proteins and consequently impair viral replication (Additional file 7B). For the D19A site, following the insertion of an exogenous expression cassette lacking the SV40 poly(A) termination signal, robust eCMV transcription produces extended transcripts that disrupt alternative splicing of ORF16/ORF17 and reduce their expression (Additional file 7C). Notably, deletion of ORF16/ORF17 has been shown to impair viral replication [26], suggesting these proteins act as important auxiliary factors. By eliminating such read-through interference, the SV40 poly(A) terminator thus preserves proper expression of ORF16/ORF17. This may primarily account for the faster CPE development and higher viral titer of rD19A_CRS (containing the SV40 poly(A) terminator) compared with rD19A_CR.

Notably, the exogenous insertion at the D19A site showed poorer genetic stability when the termination signal was omitted, under the same genomic background. We propose that one potential mechanism is that intense eCMV-driven transcription generates excessive transcripts, which may form R-loops with genomic DNA, trigger host homologous repair, and ultimately lead to the loss of the exogenous ORF [38, 39]. Collectively, the design of exogenous gene expression cassettes affects recombinant viral replication phenotypes by modulating native viral gene expression, which further impacts transgene expression and genetic stability. Our findings highlight the complex, site-dependent interplay between transcriptional regulatory elements (such as the eCMV promoter and SV40 poly(A) terminator) in the development of FAdV-4-based vectors.

In summary, by integrating a detailed transcriptional map with systematic experimental characterization, this study reveals that the design of exogenous gene expression cassettes can profoundly modulate the native transcriptional landscape of FAdV-4, thereby altering viral replication and genetic stability. These mechanistic insights guided the strategic integration of the D14AB and D19A sites to construct a promising backbone for multivalent attenuated vaccine development. However, this study was limited to the stable expression of model proteins (fluorescent proteins of different emission colors), which are generally stable, simple in structure, and easy to express. In contrast, authentic exogenous viral proteins—such as the Newcastle disease virus F protein or infectious bursal disease virus VP2 protein—possess complex spatial structures. Their expression products often involve intricate post-translational modifications, conformational changes, and self-assembly [40–42]. Thus, model proteins cannot fully recapitulate the expression, processing, or immunogenicity of real viral antigens.

To fully explore the translational potential of our vector system within the insertion capacity constraints, future studies will express the NDV F gene (1662 bp) at the D14AB site (max 2349 bp) and an optimized minimal immunogenic fragment of IBDV VP2 at the D19A site (max 1257 bp). Recombinant viruses will be used to immunize SPF chickens, followed by systematic evaluation of antigen-specific immunogenicity, safety, and protective efficacy. These efforts will further validate the utility of our dual-site vector platform and expand its application for the development of promising multivalent avian vaccines.

## Supplementary Information

**Additional file 1. List of primers.** Primer sequences used in this study are provided in this Additional file.

**Additional file 2. Schematic diagram of different fluorescent protein expression cassettes.** (**A**) Schematic diagram of the composition of a single fluorescent protein expression cassette. The promoter is an enhanced CMV promoter, and the transcriptional termination signal uses the SV40 polyadenylation signal. (**B**) Schematic diagram of the composition of multiple fluorescent protein expression cassettes. The promoter is an enhanced CMV promoter, and the transcriptional termination signal uses the SV40 polyadenylation signal. In the dual-fluorescent expression cassette, the ORFs of the two fluorescent proteins are connected by the Foot-and-mouth disease virus 2A self-cleaving peptide (F2A peptide); in the triple-fluorescent expression cassette, the ORFs of the three fluorescent proteins are alternately connected by the F2A peptide and Porcine teschovirus-1 2A self-cleaving peptide (P2A peptide).

**Additional file 3. Construction of recombinant FAdV-4 viruses expressing single fluorescent protein at D2042 site.** (**A**) Schematic diagram illustrating the construction of recombinant FAdV-4 strains. (**B**) Rescue of single-fluorescent-protein recombinant FAdV-4 strains. At 48 hours and 96 hours post-transfection with the infectious clone plasmids, the expected color of fluorescence was observable for all single-fluorescent recombinant viruses under an inverted fluorescence microscope. (**C**) Passage stability of recombinant FAdV-4 strains with or without the SV40 poly(A) terminator (Only data on the passage with the best fluorescent expression effect and the corresponding observation time in each recombinant virus were presented). One-step growth curves of rD2042_CG strains determined by viral titer and viral DNA copy numbers, respectively. All data in this figure are presented as the mean ± SEM (n = 3). Statistical differences in data were analyzed by two-way ANOVA followed by Dunnett’s multiple comparisons test (*P <0.05, **P <0.01, ***P <0.001, ****P <0.0001 vs. the rFAdV-4 group).

**Additional file 4. PCR analysis of genetic stability at the D14AB (A) and D19A (B) sites in the ra14RG19B strain.** Lanes 1–6: DNA extracted from the ra14RG19B strain at passages 10 to 15 was used as the amplification template, respectively. Lane 7: Infectious clone plasmid pD14AB was used as the amplification template. Lanes 8 and 10: Infectious clone plasmid pra14RG19B was used as the amplification template. Lane 9: Infectious clone plasmid pD14AB was used as the amplification template.

**Additional file 5. Sequencing results of D14AB and D19A insertion sequences in P10 and P15 ra14RG19B strain.** The raw sequencing data for the exogenous inserts at the D14AB and D19A sites (passage 10) and the D14AB site (passage 15) of ra14RG19B are provided in Additional file 5.

**Additional file 6. Safety evaluation of passage-10 ra14RG19B (challenge dose: 10⁷·⁰ TCID₅₀/bird; n=5)**. Fourteen-day-old chickens were challenged intramuscularly and observed for 14 days. The CH-JS-2017 group received the highly virulent clinical FAdV-4 strain CH-JS-2017; the ra14RG19B group received the 10th-passage triple-fluorescent recombinant virus ra14RG19B (attenuated rON1 backbone); the Mock group received an equal volume of DMEM/F12.

**Additional file 7. Schematic diagram of transcription units at and adjacent to the D2042 (A), D14AB (B), and D19A sites (C).** Blue arrows represent native ORFs of the viral genome. Purple arrows indicate viral ORFs speculated to be affected by exogenous expression cassette insertion at each site. Black arrows denote the transcription direction of inserted exogenous cassettes. Red dashed boxes indicate the exogenous expression cassettes inserted at each site. Bold ORFs represent key replication-related genes in each transcriptional unit. Transcriptional unit 1 is controlled by the promoter at nt 35945, which initiates transcription of ORF20, ORF20A, and ORF22. Transcriptional unit 2 is regulated by multiple promoters including those at nt 35747, 37061, 39979, and 42842, driving transcription of ORF43, ORF28, ORF29, GAM1, and ORF19A. Transcriptional unit 3 is primarily governed by the promoter at nt 9275; it mediates high-level transcription of essential genes such as Hexon, as well as low-abundance transcription of ORF4 and ORF19. Transcriptional unit 4 is mainly controlled by the promoters at nt 466 and 1057, driving transcription of ORF0, ORF1, ORF1B, and ORF2.Transcriptional unit 5 is regulated by the promoters at nt 17972, 8605, and 6405, and controls expression of core replication protein ORFs, including DNA polymerase and pTP. Transcriptional unit 6 is predominantly driven by the promoter at nt 39698, responsible for transcription of ORF16 and ORF17. This schematic is adapted from the transcriptional map reported by Lu et al. [28]; only transcription units adjacent to the three insertion sites are shown for structural illustration and do not reflect actual genomic distances.

## Data Availability

All data generated during this study is provided within the manuscript.
